# Sports motivation: a narrative review of psychological approaches to enhance athletic performance

**DOI:** 10.3389/fpsyg.2025.1645274

**Published:** 2025-08-04

**Authors:** Walaa Jumah Alkasasbeh, Sofia Hwaishel Akroush

**Affiliations:** ^1^Program of Sports Management and Training, Department of Administration and Curriculum, Faculty of Arts and Educational Sciences, Middle East University, Amman, Jordan; ^2^Department of Physical and Health Education, Faculty of Education Sciences, Al-Ahliyya Amman University, Al-Salt, Jordan

**Keywords:** sports motivation, psychological approaches, intrinsic motivation, extrinsic motivation, athletic performance

## Abstract

This narrative review examines the impact of psychological motivation on athletic performance by exploring the relationship between intrinsic motivation (IM) and extrinsic motivation (EM), and their respective effects on athletes. The primary aim is to understand how different types of motivation influence performance across various sports and to highlight the role coaches play in enhancing athlete motivation. The review draws on 97 studies published between 2001 and 2024, sourced from reputable academic databases such as Google Scholar, PubMed, Scopus, and PsycINFO. Included studies specifically addressed the effects of IM and EM on athletic performance and the influence of coaching strategies. Studies unrelated to sports performance or motivation were excluded. Findings indicate that intrinsic motivation rooted in personal goals, enjoyment, and self-determination has a more sustained and profound impact on athletic performance, promoting long-term commitment and continuous improvement. In contrast, extrinsic motivation driven by rewards, recognition, or social pressure tends to produce short-term gains but lacks enduring influence. The review underscores the pivotal role of coaches in fostering motivation by providing individualized feedback, cultivating a supportive and positive environment, and recognizing athlete efforts. Furthermore, a comparison between individual and team sports suggests that IM is more prevalent and influential in individual sports, whereas EM and social reinforcement play a larger role in team-based settings. The review concludes that a strategic combination of intrinsic motivation, external reinforcement, and a supportive social climate is essential for sustained athletic success. Coaches and sports programs should integrate these elements when designing motivational approaches tailored to their athletes’ needs.

## Introduction

Psychology focuses on studying the nature of behavior, its functions, and mental experiences (Colman, 2015). Sport and exercise psychology systematically examines the behaviors, thoughts, and emotions of individuals engaged in sport and physical activity (Abu Jamous et al., 2024; Alkasasbeh and Amawi, 2023; Akroush et al., 2025; Amawi et al., 2025; Ohuruogu et al., 2016). Sport psychologists work with athletes and coaches to enhance performance at both professional and elite levels (Ohuruogu et al., 2016). Athletic performance is shaped by complex interplay of various factors (Soares et al., 2020). Every athlete aims to excel, achieve victory, and continuously improve their performance (Arribas Galarraga et al., 2017; Ruiz-Vanoye et al., 2017). Players put in significant effort to achieve both individual and team goals. Success in sports requires athletes to perform at their best within their capabilities to reach the highest levels of achievement (Arribas Galarraga et al., 2017). Sports performance studies have addressed the impact of various variables on performance (Abdullah et al., 2016), with psychological abilities playing a major role in enhancing it (Olmedilla et al., 2010). The psychological characteristics of players are important, not only for their direct impact on performance but also as a mediator between physical and technical competencies and athletic performance (Anderson et al., 2014; Arthur et al., 2017; Olmedilla et al., 2018; Formenti et al., 2019; Trecroci et al., 2020). Analyzing these variables contributes to improving athletes’ performance (Marco et al., 2007; Olmedilla et al., 2010). For young athletes, the perception of performance is often as important as actual performance (Soares et al., 2020).

Motivation is one of the most popular research topics in sport psychology, as it plays an important role in influencing people’s well-being and sporting performance (Orhan et al., 2025; Setyobroto, 2001). Motivation is one of the main psychological factors in improving athletic performance (Rabaz et al., 2015; Fletcher and Sarkar, 2012; Gillet and Vallerand, 2016; Gómez-López et al., 2013; MacNamara et al., 2010). Motivation can be defined as the interaction of internal and external forces that initiate, sustain, and enhance individuals’ engagement in sport and physical activity (Shoxrux, 2023). It not only drives performance but also promotes long-term adherence to exercise and competitive sports. Furthermore, motivation is closely associated with other psychological constructs such as physical self-concept and emotional intelligence. Intrinsic motivation, in particular, has been shown to correlate positively with higher levels of physical self-concept and better emotional regulation (Conde-Pipó et al., 2021). Additionally, motivation is not fixed; it is dynamic and situational. Highly self-determined individuals tend to experience a positive evolution of situational intrinsic motivation during task engagement, indicating the importance of creating supportive and autonomy-enhancing environments in sports settings (Angot and Martinent, 2025). Overall, motivation is a multidimensional and evolving construct that varies across age groups, psychological profiles, and situational contexts. This underlines its central role in both theoretical and applied domains of sport psychology research and practice (Shoxrux, 2023).

Motivation in sports is a multidimensional construct that encompasses various sources, including intrinsic factors such as personal satisfaction, extrinsic incentives like rewards, and social influences such as coaching and team dynamics. Understanding this complex nature of motivation is crucial because it directly impacts athletes’ engagement, persistence, and overall performance (Deci and Ryan, 2000). Within this framework, creativity and innovation play a significant role. These terms refer to athletes’ ability to generate novel ideas and implement new strategies during training and competition, which facilitates adaptation to changing conditions and provides a competitive advantage (Vaughan et al., 2019). Furthermore, the concept of immediate performance highlights the short-term improvements in athletic output that occur in response to motivational stimuli. This term emphasizes how quickly an athlete can enhance effort and focus following external incentives, which is critical for achieving timely competitive results (Liu et al., 2022).

According to Self-Determination Theory (SDT), motivation exists on a continuum, ranging from amotivation to extrinsic motivation (EM) and intrinsic motivation (IM) (Ryan and Deci, 2000; Deci and Ryan, 2013). EM can be classified into controlled (external and internal regulation) and autonomous forms of regulation (Lonsdale et al., 2008). IM, which is driven by inherent enjoyment and interest in the activity, represents the highest form of self-determined motivation and is associated with positive outcomes such as engagement and well-being (Ryan and Deci, 2024). Studies have demonstrated that IM can significantly improve athletic performance, even when proficiency levels are equal (Gillet et al., 2009; Gillet et al., 2012; Pope and Wilson, 2015).

According to self-determination theory, the satisfaction of three fundamental psychological needs competence, autonomy, and relatedness is what propels IM (Ryan and Deci, 2024). Competence refers to the need to feel effective and masterful, while autonomy relates to the individual’s ability to self-regulate their actions and life experiences. Relatedness refers to the feeling of social connectedness (Ryan and Deci, 2024; Ryan and Deci, 2020). When these basic needs are met, intrinsic motivation increases, contributing to improved mental readiness and athletic performance. Multiple studies have shown a relationship between the satisfaction of these needs and athletic performance (Cerasoli et al., 2016), particularly regarding competence (Sheldon et al., 2013). Several factors influence young athletes’ decision to leave sport, Including factors like lack of enjoyment, perceived competence, social pressures, competing priorities, and injuries (Molinero et al., 2006; Crane and Temple, 2015), leading to decreased performance (Oliveira et al., 2007).

Studies indicate that coaches play a significant role in influencing athletes (Almagro et al., 2010; Pineda Espejel et al., 2017; Pulido et al., 2018), as the motivational model of the coach-athlete relationship explains how coaches can impact athletes’ motivation, making them a key factor in determining performance and persistence (Mageau and Vallerand, 2003). Additionally, children’s intention to participate in sport can be a strong predictor of their motivation and behavior related to sport activity (Goudas et al., 1995). Positive experiences in sport contribute to enhancing the intention to continue physical activities (Ajzen et al., 2018), which in turn boosts commitment to sport (Abu Jamous et al., 2024). These positive experiences are associated with a strong self-concept and a high perception of competence during practice (Moreno et al., 2007). Participation rates in sports have increased, prompting researchers to study individuals’ motivations for sports participation (Cunningham and Kwon, 2003). Sport psychology aims to analyze the reasons why individuals choose or discontinue a particular sporting activity and the influence of factors such as age, gender, and experience on their motivations (Jones et al., 2006). Assessing motivations for participation also helps in understanding the priorities and strategies that support continued physical activity (Weiss and Petlichkoff, 1989). Motivations drive and direct individuals’ behavior and include intrinsic factors such as enjoyment and skill development, as well as extrinsic factors such as rewards and health improvement (Deci and Ryan, 2013). Skill development, entertainment, and fitness are among the most prominent reasons for sports participation (Deci and Ryan, 2013). Participants’ motivations in competitions differed from those in routine sports activities (Frederick and Ryan, 1993), while Ntoumanis et al. (2007) highlighted the role of enjoyment motivation as a predictor of continued participation.

Addressing sports motivation (SM) from a psychological perspective is crucial for improving athletic performance and achieving success at various levels (Kostopoulos et al., 2024). Understanding psychological motivations and their impact on athletic performance is a vital step in developing strategies to enhance players’ commitment and continuity in sports (Kostopoulos et al., 2024). This research bridges the knowledge gap regarding how psychological motivation influences players’ performance, encompassing various dimensions of sports participation, whether competitive or recreational. Highlighting the relationship between SM and other psychological factors contributes to the design of effective and integrated training programs that enhance athletes’ performance and support their sustainable development. This research also serves as an important pillar for understanding the challenges faced by young athletes, such as low enjoyment or social pressures, and for providing innovative solutions to overcome them. This narrative review aims to explore psychological approaches to enhancing performance by analyzing the role of motivation, identifying the factors influencing continued participation, and highlighting how coaches, parents, and organizations can support athletes in reaching optimal performance.

### Key questions

How does self-determination influence athletic performance across different sports disciplines?

What are the long-term effects of intrinsic versus extrinsic motivation on sustained athletic engagement?

How do coaching strategies enhance or hinder athletes’ motivation based on self-determination theory?

## Methodology

A narrative review was conducted to explore the impact of psychological motivation on athletic performance and to identify the relationship between different types of motivation (intrinsic and extrinsic) and athletes’ performance. The aim of this review was to understand how psychological motivation affects athletic performance and how coaches can develop effective motivational strategies to enhance performance. An initial search retrieved approximately 150 studies published between 2001 and 2024. After applying inclusion and exclusion criteria, a total of 97 studies were selected for detailed analysis. The literature was collected from reputable academic databases, including Google Scholar, PubMed, Scopus, and PsycINFO, as well as specialized journals in sports psychology.

### Literature selection process

**Figure 1:**
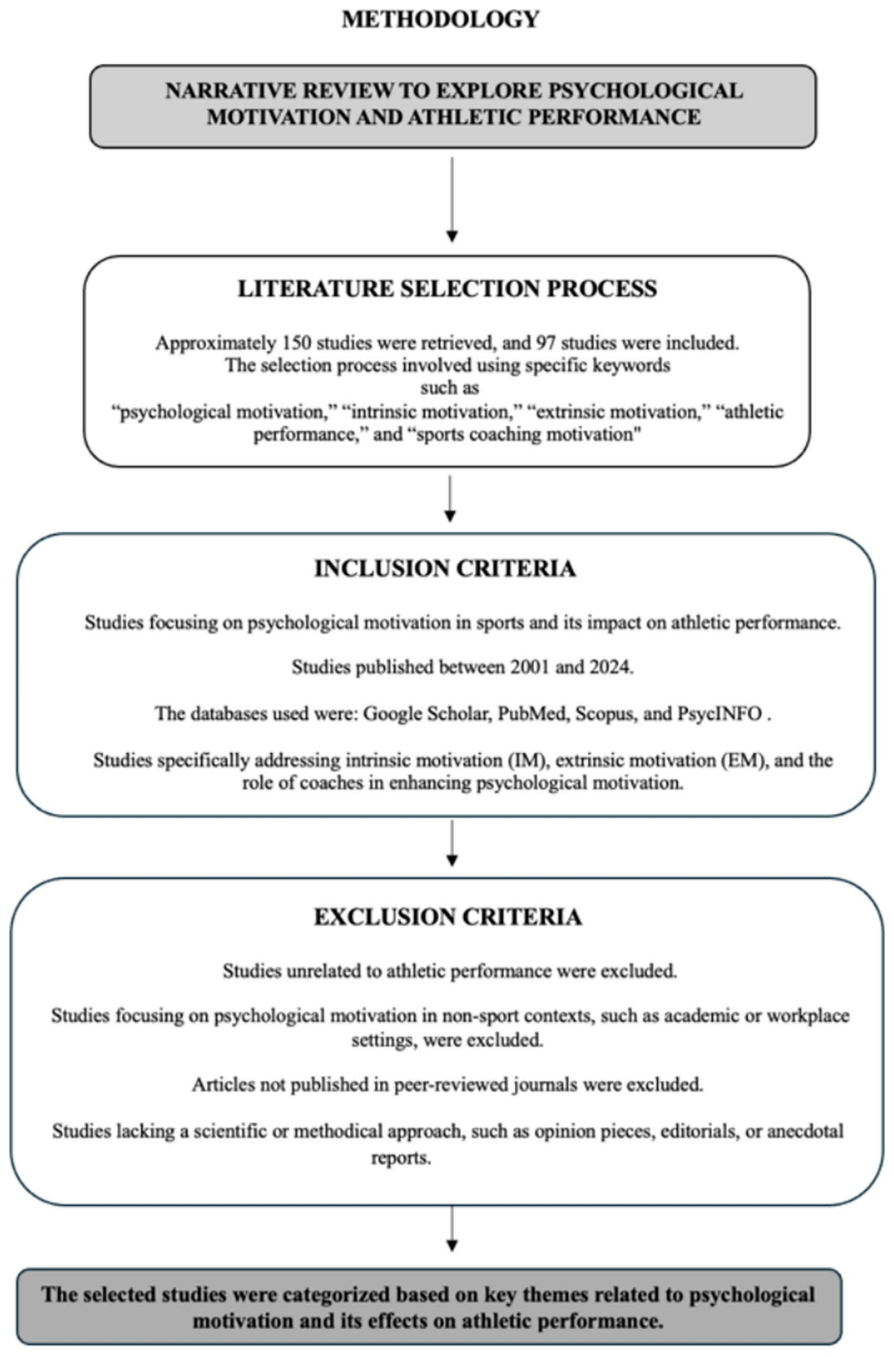
Steps of the narrative review methodology.

The literature selection process for this narrative review involved a comprehensive search using specific keywords such as “psychological motivation,” “intrinsic motivation,” “extrinsic motivation,” “athletic performance,” and “sports coaching motivation.” Titles and abstracts of the retrieved articles were initially screened to identify studies relevant to the review’s objectives. Subsequently, full texts of selected studies were reviewed to ensure they met the inclusion criteria. Included studies focused on psychological motivation within sports contexts and its impact on athletic performance, were published in peer-reviewed journals between 2001 and 2024, and specifically addressed intrinsic motivation (IM), extrinsic motivation (EM), and the role of coaches in enhancing motivation. Studies were excluded if they did not directly pertain to athletic performance, addressed motivation outside the sports context, or lacked methodological rigor, such as non-empirical or descriptive reports. This process allowed for a comprehensive synthesis of relevant literature to provide an informed understanding of psychological motivation in sport (see Figure 1).

### Presentation of the conceptual model of the study

**Figure 2:**
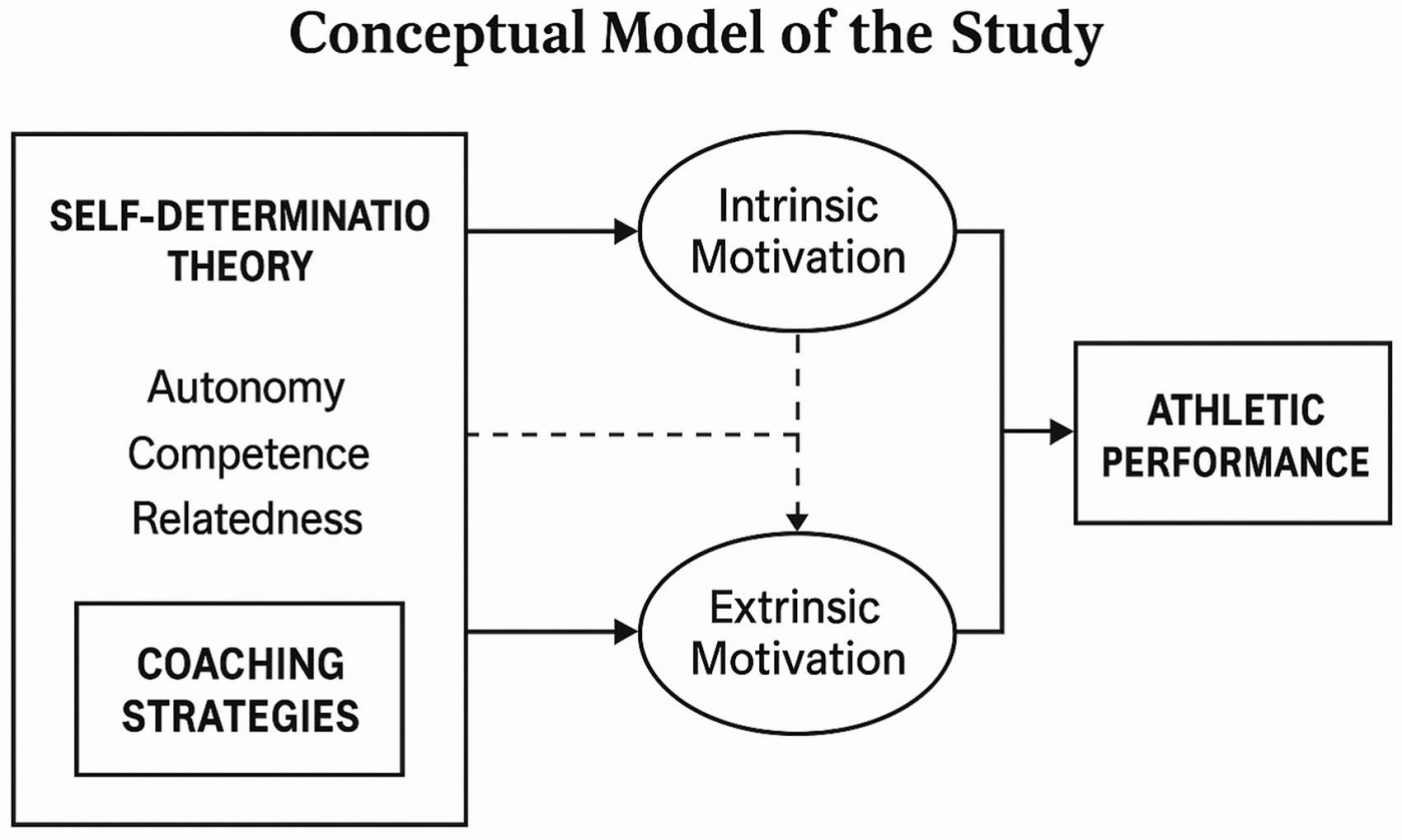
Presentation of the conceptual model of the study.

This study proposes a conceptual model illustrating the role of motivation in enhancing athletic performance. The model integrates core components of Self-Determination Theory (SDT), coaching strategies, and the dynamic interplay between intrinsic and extrinsic motivation (see Figure 2).

### IM in sports

Intrinsic motivation (IM) in sports originates from within the individual, driving them to participate in activities for self-satisfaction rather than external rewards (Pelletier et al., 2013; Djamarah, 2011). It emphasizes the quality of subjective experiences and personal goals, fostering discipline, perseverance, and independence (Brown, 2019; Coon and Mitterer, 2013). IM in sports is characterized by curiosity, skill mastery, and a drive for continuous learning (Pelletier et al., 2013; Walczak and Tomczak, 2019) enhancing athletes’ ability to set and achieve personal goals and persist through challenges (Azid et al., 2023). IM improves athletes’ discipline, adherence to training programs, and focus on goal-setting, leading to enhanced overall performance. It fosters independence, empowering athletes to make decisions autonomously and innovate in their training and performance approaches. By promoting continuous learning and resilience, IM supports athletes in pushing their limits and achieving optimal results in sports. This condensed version captures the essence of IM in sports, highlighting its benefits in enhancing athletes’ performance through self-driven motivation and personal growth.

### EM in sports

EM stems from external factors such as rewards, praise, and social recognition that drive individuals to engage in sports activities (Ryan and Deci, 2021; Brown, 2019). Athletes motivated by EM seek material or psychological rewards such as money, gifts, or verbal praise which play a key role in fostering responsibility and enhancing efficiency in achieving goals (Azid et al., 2023; Omar-Fauzee et al., 2012). In sports, EM can significantly influence performance by promoting immediate effort, competitiveness, and commitment. Incentives such as financial prizes and public recognition boost athletes’ motivation to excel and maintain consistent training. These rewards are especially effective during periods of frustration or low drive, offering short-term reinforcement and helping athletes stay engaged. However, an over-reliance on extrinsic rewards may reduce intrinsic motivation over time, potentially affecting long-term dedication and growth. Therefore, balancing EM with IM is crucial to sustaining athlete performance and ensuring lasting engagement in sports.

## Results

### Summary of key research findings

#### The concept of motivation in sports performance

##### Definition of sports motivation

Motivation: It is the process by which the sources of individual behavior, such as internal and external drivers, are activated to fulfill the need for goal accomplishment (Taylor, 1994). This process can be understood as a combination of effort and desire aimed at reaching a specific goal (Gardner, 1985). SM in particular, refers to how athletes motivate themselves to meet challenges, complete tasks, and achieve desired outcomes (Панчук et al., 2024). The significance of SM lies in its ability to influence athletic performance, as it is the key factor in helping athletes reach their objectives. Motivation is shaped by a variety of internal and external factors, including the social, educational, and family environment. Coaches play a vital role in enhancing motivation by fostering a supportive and encouraging atmosphere (Gómez Cardoso et al., 2021) SM is driven by multiple reasons for participation, such as performance, power, and affiliation, with athletes being motivated by different aspects depending on their individual needs (Elbe, 2021). These motivations can be intrinsic, driven by self-satisfaction, or extrinsic, influenced by rewards like recognition and material incentives (Azid et al., 2023). In sports, motivation comprises a set of factors that stimulate athletes’ behavior and encourage them to achieve their goals during both training and competitions. These motivations are largely shaped by the athletes’ individual needs and perspectives (Bondareva and Sabaliauskas, 2018). Moreover, SM is a complex concept that encompasses various psychological theories, including the Goal Achievement Theory and Self-Determination Theory, which explain the underlying factors that drive motivation in sports (Roberts et al., 2018).

#### The role of a coach in motivating athletes

Self-determination theory: It is stated that the motivational environment shaped by the coach directly affects athletes’ IM and participation in sport, as this environment is linked to the fulfillment of their basic psychological needs, such as autonomy, competence, and relatedness (Ryan and Deci, 2000; Howard et al., 2021). This theory assumes that the type of motivation experienced by individuals influences the emotional states of athletes, enhancing enthusiasm, vitality, and confidence (Curran et al., 2015). Studies have also shown that IM contributes to improved psychological health and well-being in athletes (Kim et al., 2020). In this context, the relationship between the coach and the athlete is crucial for meeting these psychological needs. Research has shown that coaches who promote autonomy and encourage self-actualization help improve the emotional and physical motivation of athletes, which enhances their participation and enthusiasm (Ryan and Deci, 2020; Weinstein, 2014). The results also indicate a positive relationship between coaches who foster autonomy and the fulfillment of athletes’ psychological needs (Morbée et al., 2020; Ryan and Deci, 2020). The CAR 3C model is an essential tool for understanding the coach-athlete relationship (Jowett and Ntoumanis, 2004). It includes closeness, which represents the emotional connection between the coach and athlete; commitment, reflecting the desire of both parties to maintain the relationship; and integration, which represents the interaction between them (Jowett and Ntoumanis, 2004; Choi et al., 2020). Research has also shown that a positive relationship between the coach and athletes are linked to factors such as motivation, satisfaction of psychological needs, physical self-concept, social support, and well-being (Adie and Jowett, 2010; Jowett, 2009; De Muynck et al., 2017; Jowett and Cramer, 2010; Wachsmuth et al., 2022). Based on SDT, it is essential for the coach to create a sports environment that meets athletes’ basic psychological needs. This approach contributes to their motivation and enhances their performance in sports (Graña et al., 2021; De Francisco et al., 2020).

Coaches help meet athletes’ basic psychological needs by providing a supportive environment that fosters autonomy, competence, and relatedness. To support autonomy, coaches allow athletes to make decisions and take part in setting personal goals, which strengthens their sense of ownership and control over their progress. In fostering competence, they present appropriate challenges and offer constructive feedback, motivating athletes to refine their skills and build confidence in their abilities. To enhance relatedness, coaches cultivate strong relationships with athletes, encourage open communication, and create a team culture grounded in mutual support and connection. When these basic psychological needs are fulfilled, athletes experience greater motivation, improved performance, and sustained engagement in sport.

#### Practical applications of psychological motivation in sports

Psychological motivation plays a crucial role in improving athletic performance and overall well-being in sport. Various psychological strategies can be employed to enhance motivation in athletes, contributing to better training outcomes and competitive success. Among these strategies, effective goal setting is a key component of sport psychology, helping athletes focus on clear and measurable objectives, which enhances training sessions and competitive behavior (Gearity and Gillham, 2024).

Athletes who set precise goals typically demonstrate increased motivation and commitment, leading to improved performance. Additionally, Rational Emotive Behavior Therapy (REBT) can be integrated to help athletes address irrational beliefs that may hinder their performance (Tóth et al., 2024). By replacing these beliefs with more rational ones, athletes can reduce anxiety and enhance mental resilience, ultimately improving performance in competitions. Coaches can also implement needs-supportive strategies to create an environment that fosters IM among athletes (Campbell et al., 2022). This approach emphasizes the importance of understanding athletes’ psychological needs, which enhances motivation and engagement in training. Coaches can further implement strategies to build athletes’ self-confidence, such as positive reinforcement, visualization techniques, and providing constructive feedback, which can help athletes perform better under pressure (Gearity and Gillham, 2024).

However, it is important to recognize that athletes do not respond uniformly to motivational strategies. Individual differences in personality and experience may influence the effectiveness of these techniques and how athletes respond to them.

#### Individual differences in response to motivational strategies

It is essential to recognize that athletes do not respond uniformly to motivational techniques (Roberts and Treasure, 2012). Individual differences such as personality traits, past experiences, and levels of competitivenes can influence how effective certain strategies are (Vallerand, 2007). For example, while some athletes thrive under structured goal-setting programs, others may be more motivated by autonomy-supportive environments or personal encouragement (Deci and Ryan, 2000). Understanding these individual variations allows coaches to tailor their approach, ensuring that motivational strategies are both effective and athlete-centered (Roberts and Treasure, 2012; Mallett and Hanrahan, 2004).

#### Future directions for research on sports motivation

The development of research in the field of sports motivation reflects a growing interest in understanding the factors that contribute to positive outcomes for athletes and participants. In this context, future trends in this research can be categorized into several key areas, with an emphasis on the need for a comprehensive approach to motivating athletes. In this framework, recent research indicates a shift from the Achievement Goal Theory to the Self-Determination Theory as a primary framework for understanding sports motivation (Alcaraz et al., 2014). Future research should also explore the multidimensional aspects of motivation, particularly how different types of motivation affect athletic performance and athletes’ well-being (Standage and Ryan, 2020). On the other hand, the quality of interactions between parents, coaches, and peers plays a critical role in shaping youth motivation. Positive relationships can enhance IM and make athletes enjoy sports more overall (Weiss et al., 2019). Additionally, adopting developmental perspectives can help design sports programs that better meet the needs of young athletes (Weiss et al., 2019). Regarding practical applications, translating research findings into practical strategies for coaches and sports organizations can contribute to improving the motivational climate and athletes’ outcomes (Weiss et al., 2019). Moreover, focusing on individual motivation can provide insights on how athletes can improve performance through self-motivation (Gillet and Vallerand, 2016). While research on athlete motivation is important, it is also essential to consider the negative effects that may arise in competitive environments, such as stress and burnout caused by high pressure and poor training practices. Therefore, achieving a balance between these perspectives will be vital for future research.

The findings of this narrative review emphasize the complex and multifaceted nature of motivation in sports, showing that both intrinsic and extrinsic motivations play essential roles in shaping athletes’ performance. While EM can provide short-term boosts, IM tends to have a more profound and lasting impact on athletes’ overall development and performance. Coaches, by creating supportive and personalized environments, can play a pivotal role in fostering both types of motivation, tailoring their approaches based on the individual needs of athletes. Moreover, the distinction between individual and team sports in terms of motivation is crucial. Athletes in individual sports tend to rely more on intrinsic motivation, driven by personal achievement and self-improvement, while team sports athletes often benefit from external motivations like rewards and team dynamics. This indicates that motivational strategies should be sport-specific, taking into account the unique demands and structures of each sport. The findings also highlight the importance of the long-term benefits of intrinsic motivation. Although extrinsic rewards can enhance performance in the short run, they may not foster a sustainable commitment to the sport. Long-term success in athletics requires fostering a sense of self-determined motivation, where athletes find personal meaning and satisfaction in their involvement, beyond external rewards. Additionally, the role of the environment, including social support from coaches, teammates, and family, was emphasized. A positive, motivating environment can act as a buffer during challenging times and help athletes stay committed and focused on their goals. In conclusion, psychological motivation is a key factor in athletic performance, and effective motivational strategies must consider both intrinsic and extrinsic motivators. Coaches play an essential role in fostering motivation, not only by providing rewards but also by cultivating an environment that encourages self-determination, personal growth, and a sense of belonging. Understanding the dynamics of motivation in different sports and contexts will enable coaches to implement tailored strategies that enhance athletes’ performance and overall well-being.

## Conclusion

This review highlights the vital role of psychological motivation in improving athletic performance. Intrinsic motivation rooted in self-determination and personal goals supports long-term commitment, skill growth, and resilience. Extrinsic motivation, like rewards and external feedback, offers short-term performance gains but works best when combined with intrinsic drives for lasting success. Coaches are key in creating balanced, supportive environments that meet athletes’ individual and team needs. For example, in endurance sports, setting personal goals fosters intrinsic motivation, while in team sports, recognition and positive feedback boost extrinsic motivation. Addressing challenges such as injury or performance slumps requires adaptive motivational strategies tailored to each athlete’s situation. Future research should focus on innovative motivational techniques and their effects across different sports levels. These insights can help coaches and sports programs develop more effective methods for enhancing performance and athlete well-being. Therefore, it is essential for researchers, coaches, and sports institutions to collaborate in implementing evidence-based motivational strategies that promote both high-level performance and long-term athlete development.
